# Prolyl 4-hydroxylase alpha 1 protein expression risk-stratifies early stage colorectal cancer

**DOI:** 10.18632/oncotarget.27491

**Published:** 2020-02-25

**Authors:** Atsushi Tanaka, Yihua Zhou, Jinru Shia, Fiona Ginty, Makiko Ogawa, David S. Klimstra, Ronald C. Hendrickson, Julia Y. Wang, Michael H. Roehrl

**Affiliations:** ^1^ Department of Pathology, Memorial Sloan Kettering Cancer Center, New York, NY, USA; ^2^ Department of Pathology, Graduate School of Medicine, University of Tokyo, Tokyo, Japan; ^3^ Human Oncology and Pathogenesis Program, Memorial Sloan Kettering Cancer Center, New York, NY, USA; ^4^ ICU Department, Second Affiliated Hospital of Nanchang University, Nanchang, China; ^5^ GE Global Research Center, Niskayuna, NY, USA; ^6^ Sloan Kettering Institute, Memorial Sloan Kettering Cancer Center, New York, NY, USA; ^7^ Curandis, New York, NY, USA

**Keywords:** P4HA1, colorectal cancer, biomarker, prognosis, pathology

## Abstract

Colorectal cancer (CRC) is one of the most prevalent and lethal malignancies. Especially for early stage CRC, prognostic molecular markers are needed to guide therapy. In this study, we first extracted total proteomes from matched pairs of fresh cancer and benign mucosal tissues from 22 CRC patients. Global proteomic profiling with Fourier transform liquid chromatography-mass spectrometry sequencing and label free quantitation uncovered that P4HA1 (prolyl 4-hydroxylase alpha 1) was overexpressed in CRC relative to benign colonic mucosa. We then investigated expression by immunohistochemical staining with P4HA1-specific antibodies using CRC tissue microarrays. Independent validation cohorts of 599 cases of early stage CRC and 91 cases of late stage CRC were examined. Multivariate and univariate survival analyses revealed that high expression of P4HA1 protein was an independent poor prognostic marker for patients with early stage CRC, especially of the microsatellite stable subtype. Our study provides strong support for P4HA1 as a predictive protein marker for precision diagnostics, therapeutic decision-making, and drug development for early stage colorectal cancer and demonstrates the utility of proteomic profiling to identify novel protein biomarkers.

## INTRODUCTION

Colorectal cancer (CRC) is one of the most prevalent malignant tumors and the third leading cause of cancer deaths worldwide. Despite intensive screening efforts, 30–40% of CRC patients have already developed locally advanced disease or harbor metastases when diagnosed [1]. When CRC is discovered at an early stage and the tumors are resected completely, 5-year overall survival is around 90% [2, 3]. Risk assessment of stage II CRC is particularly critical because it determines whether adjuvant chemotherapy should be administered or not. Currently, risk assessment at early stage is challenging because of a lack of reliable prognostic molecular biomarkers. Morphological features such as poorly differentiated histology, lymphovascular invasion, bowel obstruction, perineural invasion, localized perforation, and positive margins have been reported to worsen the prognosis of stage II CRC [4–6]. However, molecular biomarkers with more precise prognostic value, preferably with an underlying functional pathophysiologic rationale, are needed, as such markers would enable us to better stratify risk of recurrence in resected early stage CRC after resection and more accurately select patients for adjuvant therapy, while avoiding overtreatment in low-risk early stage CRC.

While numerous genomic and transcriptomic studies have been performed, these have resulted in disappointingly few protein-based biomarkers [7]. This may be explained by the low global concordance between mRNA abundance and protein expression levels in human CRC [8]. Similar RNA-protein discordance has been observed in yeast, mouse, and human cell lines [9–11]. We can overcome this limitation by directly analyzing the global protein expression profiles in human patient tissues. Proteomics with latest-generation liquid chromatography-mass spectrometry (LC-MS) can detect 5,000–10,000 proteins in one shotgun sequencing event, and such powerful and sensitive technology may enable us to discover prognostic protein biomarkers for early stage CRC that previous genomic and transcriptomic analyses would have missed. Combining results from 712 patients, our study shows that collagen prolyl 4-hydroxylase alpha 1 (P4HA1) protein expression robustly risk-stratifies early stage CRC.

## RESULTS

### Differential protein expression analysis of colorectal cancer tissues

**Figure 1:**
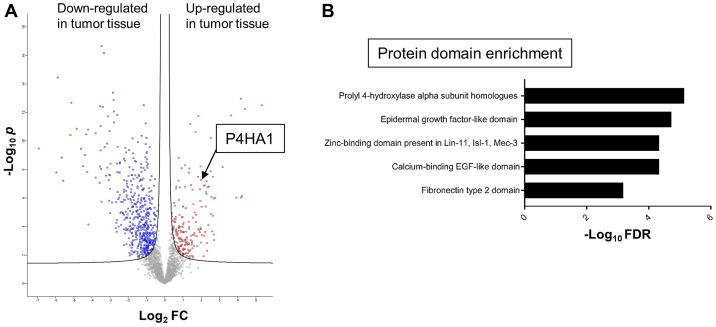
Mass spectrometric proteomics of CRC and global protein domain enrichment analysis. (**A**) Volcano plot of relative abundances of proteins from CRC relative vs. benign colonic mucosa as measured by mass spectrometry in matched samples from 22 patients. Among a total of 2,949 proteins displayed in the plot, we found 730 significantly differentially expressed proteins including 197 (red) up- and 533 (blue) down-regulated proteins. The hyperbolic solid lines show the false discovery rate frontier (FDR) set to 0.01. The x-axis shows the log_2_ of the fold change (FC) of protein abundance (ratio of cancer to benign mucosa). The y-axis shows the negative log_10_ of the *t*-test *p* value for a particular protein (dot in the volcano plot). (**B**) Global protein domain enrichment analysis of CRC up-regulated proteins using the Simple Modular Architecture Research Tool (SMART).

To discover potential biomarkers for CRC, our first goal was to identify proteins that are differentially expressed in tumor tissue, particularly those that are over-expressed in tumors relative to benign colonic mucosa. For optimal signal, we chose cancer tissue samples that had high tumor content, minimal necrosis, and minimal blood contamination. A total of 6,638 proteins were identified from all tissue samples, and 2,949 proteins were found to be shared by 70% or more of samples. To find differentially expressed proteins in CRC vs. benign colonic mucosa, *t*-tests with 1% false discovery rate were performed, resulting in 197 up-regulated and 533 down-regulated proteins in tumor tissues, respectively (Figure 1A). Reassuringly, several known CRC biomarkers, such as S100A9 and Tenascin-C, were found to be overexpressed in the tumor tissues by our mass spectrometric approach [12–14].

A computational protein domain/peptide sequence enrichment analysis revealed as the top 5 among the 197 up-regulated proteins the following: prolyl 4-hydroxylase alpha subunit homologues, epidermal growth factor-like domains, zinc-binding domains, calcium-binding EGF-like domains, and fibronectin type 2 domains (Figure 1B). Interestingly, prolyl 4-hydroxylase alpha subunit homologues, which include P4HA1, P4HA2, P3H1, PLOD1, PLOD2, and PLOD3 (all of which were detected in our LC-MS data), emerged as the top enriched domain/sequence. We selected P4HA1 for further investigation because (i) P4HA1 showed the highest expression level among these 6 proteins in CRC tissues, (ii) P4HA1 overexpression has shown positive correlation with tumor progression in breast cancer, prostate cancer, and high-grade glioma [15–17], and (iii) prognostic relevance of P4HA1 in CRC has not been studied.

### Validation of P4HA1 expression in CRC patients

**Figure 2:**
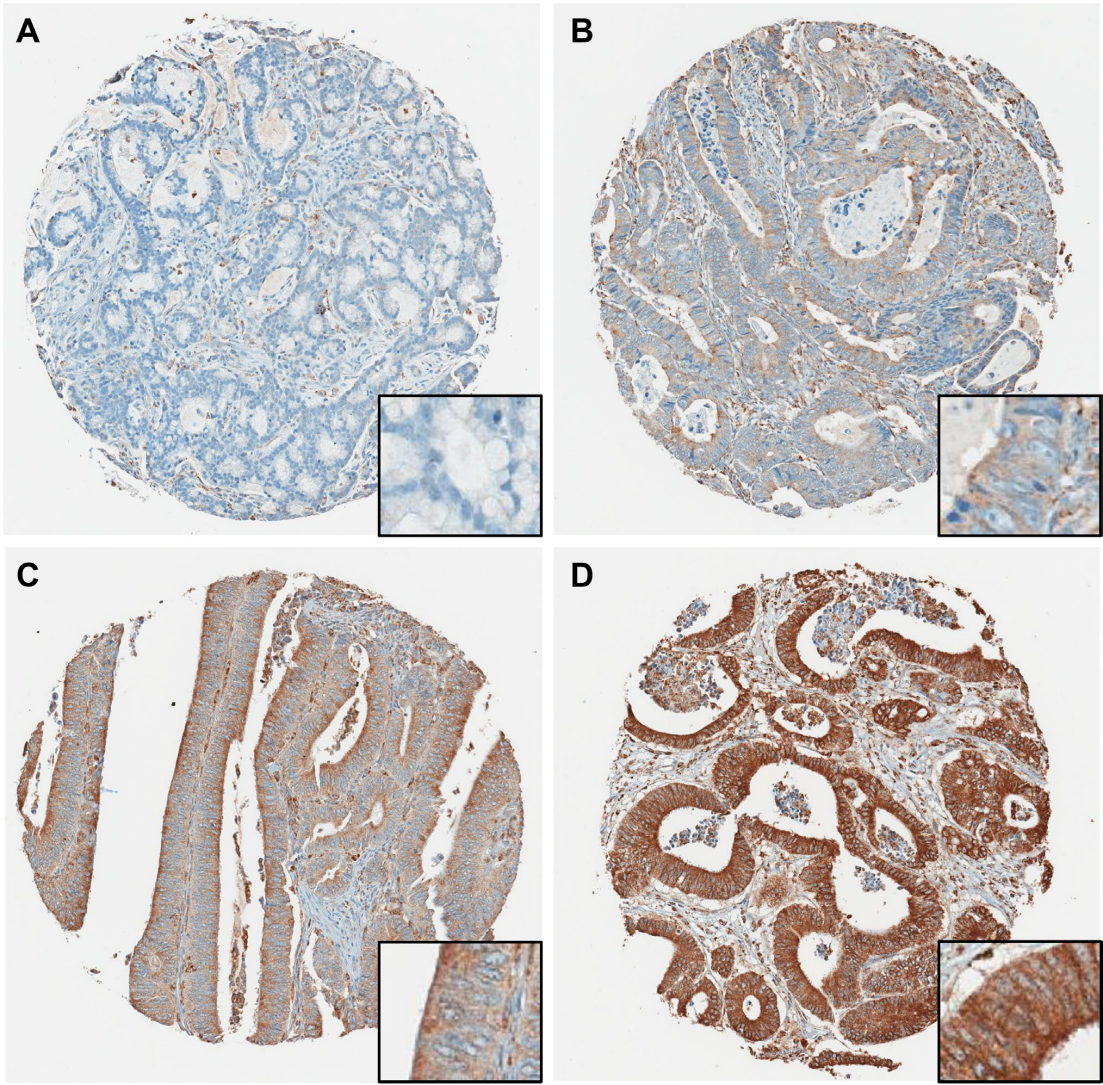
Representative P4HA1 immunohistochemical staining showing four different TMA cores. (**A**) Negative (0), (**B**) weakly (1+) positive, (**C**) moderately (2+) positive, (**D**) strongly (3+) positive staining. Magnifications: 40× (insets, 400×).

We examined the expression of P4HA1 in CRC in a large independent validation cohort by immunohistochemistry (IHC). We first examined 599 clinical cases from 305 male and 294 female patients with stage I or II colorectal cancer (Table 1). Tissue microarrays were assembled and were probed with P4HA1-specific polyclonal antibodies. Representative IHC staining patterns are shown in Figure 2. Across the entire cohort, we observed a continuum of protein expression intensities in CRC, ranging from no expression (score, 0; Figure 2A), weak expression (score 1+; Figure 2B), moderate expression (score, 2+; Figure 2C), to strong expression (score, 3+; Figure 2D).

**Table 1:** Clinicopathological characteristics of the early stage CRC cohort

Feature	P4HA1 (*n* = 599)		*p*-value*
	Low	High	
Total	417 (69.6%)	182 (30.4%)	
Gender			0.8570
Male	205 (67.2%)	100 (32.8%)	
Female	212 (72.1%)	82 (27.9%)	
Age (years)			0.2137
≤65	174 (70.2%)	74 (29.8%)	
>65	243 (69.2%)	108 (30.8%)	
Histology			>0.9999
Mucinous	31 (68.9%)	14 (31.1%)	
Not mucinous	386 (66.4%)	168 (30.3%)	
Tumor differentiation			0.0084
G1/G2	392 (71.1%)	159 (28.9%)	
G3	25 (52.1%)	23 (47.9%)	
Location			0.0025
Left	224 (75.4%)	73 (24.6%)	
Right	193 (63.9%)	109 (36.1%)	
TNM stage			<0.0001
I	184 (83.6%)	36 (16.4%)	
II	223 (60.4%)	146 (39.6%)	
MMR			<0.0001
Intact (MSS)	343 (74.4%)	118 (25.6%)	
Lost (MSI)	74 (53.6%)	64 (46.4%)	

MMR, mismatch repair; *Fisher’s exact test.

As expected from the functional role of P4HA1, the enzyme is expressed in the cytoplasm of epithelial cells. When P4HA1 is expressed in a particular CRC, it appears to be present rather uniformly without significant spatial heterogeneity of expression. Furthermore, P4HA1 protein expression is primarily present in the malignant epithelial component of a CRC. In some cases (Figure 2D), a subpopulation of stromal fibroblasts expresses P4HA1, suggesting hypoxia-induced matrix remodeling [18], whereas inflammatory cells are typically negative or only weakly positive. Normal benign colonic mucosa is negative for IHC-detectable P4HA1.

### Clinicopathological analysis of P4HA1 in CRC cohort

To explore the correlation of P4HA1 expression with clinicopathological features of CRC, we examined all 599 early stage cases and calculated an IHC H-score for each case. We then divided the cohort into two groups using a score threshold of 130, which corresponds to the upper 75th percentile (upper quartile) of the H-score distribution for the cohort. The cohort of 599 cases was divided into two groups, with 182 cases (30.4%) in the high-expression group with H-scores ≥130 and 417 cases (69.6%) in the low-expression group with H-scores <130 (Table 1).

As shown in Table 1, P4HA1 expression levels were compared for various clinicopathological features. There were no statistically significant differences in P4HA1 expression levels between male and female CRC patients, older and younger patients, or mucinous or not mucinous tissues. High P4HA1 protein expression was more frequently found in patients with poor (G3) tumor differentiation (*p* = 0.0084), mismatch repair loss (*p* < 0.0001), and right-sided location (*p* = 0.0025). In addition, CRC of stage II showed significantly higher P4HA1 expression than CRC of stage I (*p* < 0.0001).

### Survival time vs. P4HA1 expression

**Figure 3:**
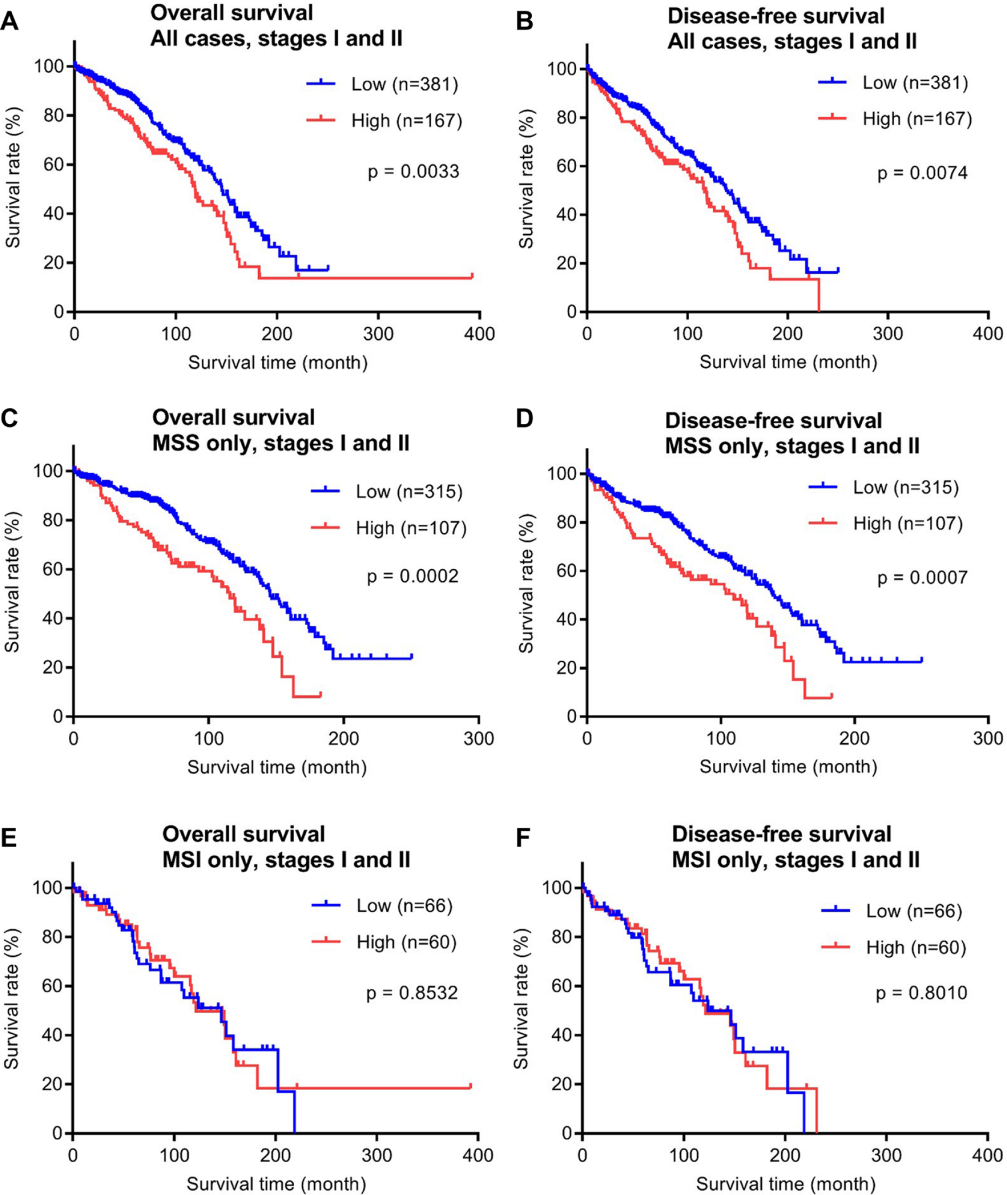
Overall survival (OS) and disease-free (DFS) survival analyses of the early stage (stages I and II) CRC validation cohort (*n* = 548) stratified by P4HA1 protein expression. (**A**, **B**) Kaplan-Meier curves with all CRC cases are shown. MSS subtype (*n* = 422) and MSI subtype (*n* = 126) analyses are shown in (**C**–**F**). The separation between low (blue) and high (red) P4HA1 expression corresponds to the 75th percentile (upper quartile) of the H-score distribution.

To evaluate the prognostic potential of P4HA1 for early stage colorectal cancer, we examined the relationship between patient survival time and P4HA1 expression using Kaplan-Meier analysis (Figure 3). Of the 599 cases examined by immunohistochemistry, 548 cases had available survival data, had been treated with surgery alone (no adjuvant therapy), and were thus used in this particular analysis (mean follow-up, 80.5 months; range, 0.2–392.5 months). Both overall survival (OS) and disease-free survival (DFS) times were analyzed. Overall, the P4HA1-high expression group showed significant shorter OS and DFS times (*p* = 0.0033 and *p* = 0.0074, respectively; Figure 3A, 3B).

Next, we analyzed the correlation between survival time and P4HA1 expression in CRC patients with microsatellite stable (MSS) or microsatellite instable (MSI) status. MSI CRC has been found to have a favorable survival rate compared with MSS CRC [19]. In our study cohort with survival data (*n* = 548), 422 patients had MSS tumors and 126 patients had MSI tumors. In cases of MSS cancer, the P4HA1-high group showed significantly shorter OS and DFS times (*p* = 0.0002 and *p* = 0.0007, respectively; Figure 3C, 3D). By contrast, in cases of MSI cancer, P4HA1 expression did not significantly correlate with OS or DFS times (Figure 3E, 3F).

**Figure 4:**
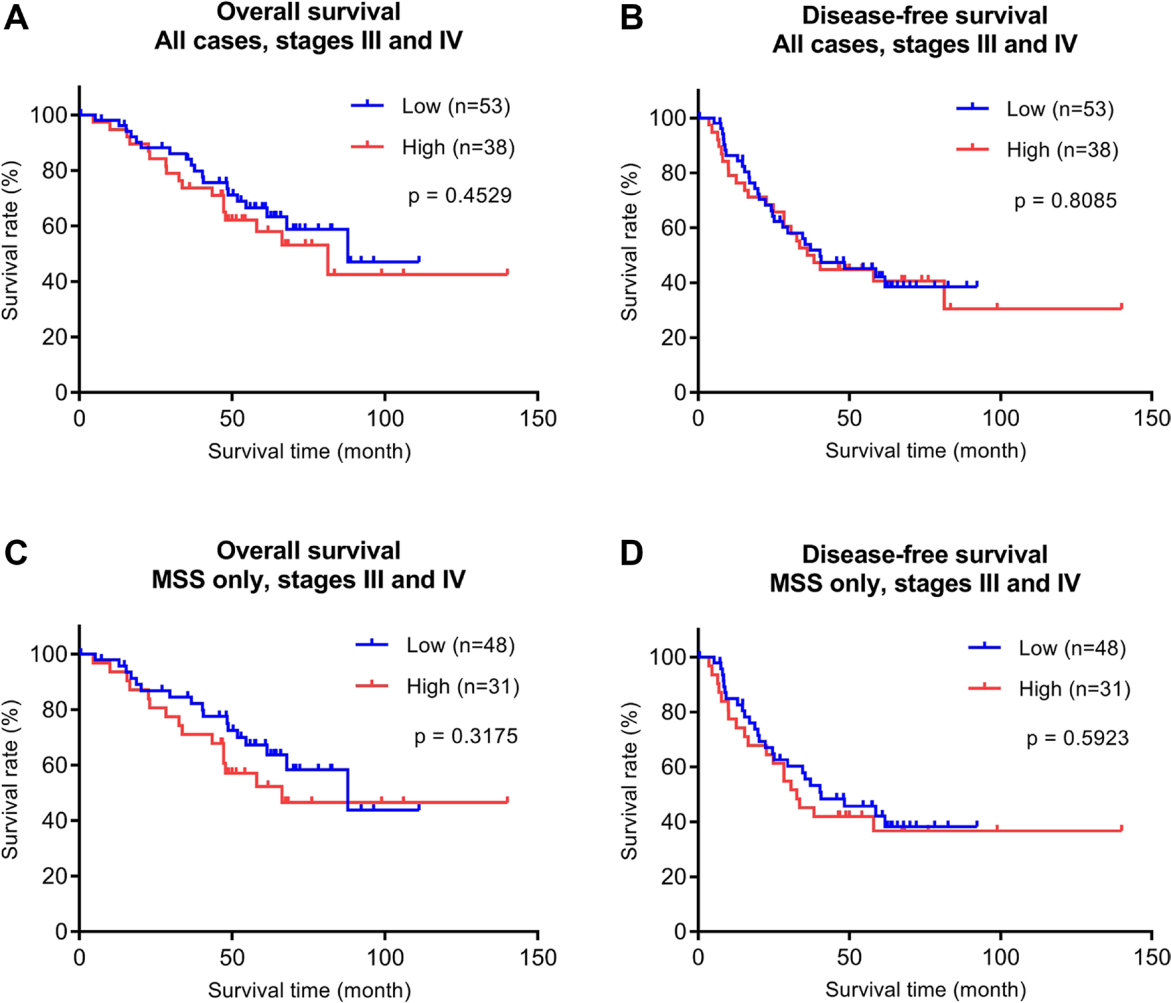
Overall survival (OS) and disease-free (DFS) survival analyses of the late stage (stages III and IV) CRC validation cohort (*n* = 91) stratified by P4HA1 protein expression. (**A**, **B**) Kaplan-Meier curves with all CRC cases are shown. MSS subtype analyses are shown in (**C**, **D**). The separation between low (blue) and high (red) P4HA1 expression corresponds to the 75th percentile (upper quartile) of the H-score distribution.

The above analysis of early (stages I and II) CRC revealed high P4HA1 expression as a poor prognostic maker in early stage MSS CRC. We then asked whether P4HA1 expression plays a similar role in late stage CRC and obtained another cohort of 91 cases with late stage CRC (stages III and IV; Figure 4). Clinicopathological features of this cohort are shown in Supplementary Table 1 (mean follow-up, 52.9 months; range, 0.4-140.0 months). Similar to the above early stage studies, we examined P4HA1 expression in these cases by immunohistochemistry, H-scoring, and statistical analyses. The differences between survival times and P4HA1 expression levels were not statistically significant in late stage CRC. Nevertheless, the P4HA1-high group showed a trend for slightly worse OS (Figures 4A and 4C).

To test whether P4HA1 expression is an independent prognostic factor for all early stage CRCs (Table 2) or only early stage MSS CRC (Table 3), we performed univariate and multivariate analyses. When all CRC cases that include MSS and MSI subtypes are evaluated, age, tumor stage, and P4HA1 expression were found to be independent predictors for OS time. However, for DFS time, only age and tumor stage were independent predictors. When only the CRC MSS subtypes were evaluated, tumor stage and P4HA1 expression were independent predictors for both OS and DFS times. Hence, these statistical analyses support the notion that high P4HA1 expression is an independent prognostic marker for poor survival in early stage CRC.

**Table 2:** Univariate and multivariate analyses of early stage CRC for correlations with survival (*n* = 548)

Variables	Overall survival				Disease-free survival			
	Univariate		Multivariate		Univariate		Multivariate	
	HR (95% CI)	*p*-value	HR (95% CI)	*p*-value	HR (95% CI)	*p*-value	HR (95% CI)	*p*-value
Gender (male vs. female)	1.18 (0.90–1.55)	0.2427			1.26 (0.97–1.64)	0.0811		
Age (years) (>65 vs. ≤65)	3.61 (2.55–5.26)	<0.0001	3.62 (2.55–5.27)	<0.0001	2.87 (2.10–4.01)	<0.0001	2.81 (2.05–3.94)	<0.0001
Tumor location (right vs. left)	1.31 (1.00–1.73)	0.0499			1.24 (0.95–1.61)	0.1124		
Histology (mucinous vs. other)	0.63 (0.34–1.07)	0.0939			0.63 (0.35–1.04)	0.0752		
Tumor differentiation (G3 vs. G1/2)	1.26 (0.72–2.03)	0.3937			1.17 (0.69–1.86)	0.5424		
AJCC stage (II vs. I)	1.72 (1.28–2.33)	0.0002	1.48 (1.09–2.04)	0.0120	1.73 (1.31–2.31)	0.0001	1.66 (1.25–2.21)	0.0004
MMR (lost vs. intact)	1.07 (0.78–1.46)	0.6655			1.00 (0.73–1.35)	0.9931		
P4HA1 expression (high vs. low)	1.52 (1.14–2.02)	0.0045	1.41 (1.04–1.91)	0.0266	1.45 (1.10–1.90)	0.0095		

Cox proportional hazards model. HR, hazard ratio; CI, confidence interval; MMR, mismatch repair.

**Table 3:** Univariate and multivariate analyses of the MSS subtype of early stage CRC for correlation with survival (*n* = 422)

Variables	Overall survival				Disease-free survival			
	Univariate		Multivariate		Univariate		Multivariate	
	HR (95% CI)	*p*-value	HR (95% CI)	*p*-value	HR (95% CI)	*p*-value	HR (95% CI)	*p*-value
Gender (male vs. female)	1.34 (0.97–1.85)	0.0732			1.44 (1.07–1.98)	0.0163		
Age (years) (>65 vs. ≤65)	3.56 (2.38–5.54)	<0.0001	3.49 (2.33–5.44)	<0.0001	2.77 (1.93–4.07)	<0.0001	2.7 (1.89–3.98)	<0.0001
Tumor location (right vs. left)	1.25 (0.91–1.72)	0.1651			1.23 (0.90–1.66)	0.1902		
Histology (mucinous vs. other)	0.71 (0.32–1.35)	0.311			0.75 (0.36–1.39)	0.3858		
Tumor differentiation (G3 vs. G1/2)	1.65 (0.51–3.91)	0.3606			1.46 (0.45–3.46)	0.4800		
AJCC stage (II vs. I)	1.84 (1.31–2.63)	0.0004	1.56 (1.09–2.26)	0.0146	1.88 (1.36–2.64)	0.0001	1.65 (1.18–2.35)	0.0036
P4HA1 expression (high vs. low)	1.9 (1.33–2.66)	0.0005	1.64 (1.14–2.34)	0.0086	1.75 (1.25–2.42)	0.0013	1.48 (1.04–2.07)	0.0282

Cox proportional hazards model. HR, hazard ratio; CI, confidence interval, MMR: mismatch repair.

## DISCUSSION

P4HA1 (prolyl 4-hydroxylase alpha 1), also known as procollagen-proline 2-oxoglutarate 4-dioxygenase alpha 1), is a member of the tetrameric α-ketoglutarate-dependent dioxygenase enzyme family [20, 21]. These enzymes catalyze the incorporation of oxygen into organic substrates. P4HA1 catalyzes 4-hydroxylation of proline in -X-Pro-Gly- motifs in diverse protein substrates [21]. The best-known substrate is collagen, and P4HA1 modification of proline to 4-hydroxyproline is essential for the proper three-dimensional folding of newly synthesized procollagen chains. Other potential substrates of P4HA1 include complement C1q, elastin, prion protein, and Argonaute 2 [21]. Hence, P4HA1 may play many important roles in various biological functions.

Up-regulation of P4HA1 has been reported in some other cancers. In melanoma, collagen P4H enzymes are reported to be bifunctional growth and tumor invasiveness regulators, and P4H family members, including P4HA1, were found to be overexpressed and associated with poor clinical outcomes [22]. In oral squamous cell carcinoma, a high *P4HA1* mRNA level was reported to be a single-gene surrogate of hypoxia and an independent prognostic marker for locoregional recurrence and OS [23]. In high-grade gliomas, high expression of P4HA1 was correlated with aggressiveness [16]. In prostate cancer, P4HA1 expression levels were associated with disease progression [15]. In triple-negative breast cancer, P4HA1 expression was induced and correlated with short relapse-free survival whether or not patients had received chemotherapy [17]. In addition, in a human protein atlas database for normal and cancer tissues [24], high *P4HA1* mRNA expression showed poorer prognosis in renal, head and neck, cervical, pancreatic, lung, and breast cancers. Recently, P4HA1 protein in blood plasma was described as part of a 4-protein panel that can differentiate patients with CRC from healthy controls [25].

Since KRAS mutations occur frequently in colorectal cancer, we asked whether KRAS mutation enrichment in the P4HA1-high group may contribute to poor prognosis in early stage CRC. We analyzed mRNA sequencing data and clinical information from the TCGA (244 CRC cohort reported in 2012) by accessing the cBioPortal for Cancer Genomics (https://www.cbioportal.org/) [26]. However, KRAS mutation status had no significant correlation with *P4HA1* mRNA expression in early stage CRC (Supplementary Figure 1). KRAS mutation status also did not show significant difference in the MSS subgroup nor in the MSI subgroup.

Recently, P4HA1 was shown to play an essential role in breast cancer tumorigenesis and distant metastases by stabilizing HIF-1α via reducing its proline hydroxylation, resulting in escape from degradation [17]. HIF-1α overexpression in CRC is related to poor prognosis, short time to recurrence, and short OS time [27–30]. We therefore wondered about a correlation of P4HA1 with HIF-1α in CRC. Examining mRNA sequencing data and clinical information from the TCGA (same cBioPortal cohort as above), we found that the mRNA levels of P4HA1 and HIF-1α in CRC were positively correlated (Supplementary Figure 2). However, at proteomic level, we were only able to reliable detect P4HA1 protein in CRC tissues by mass spectrometry. This discrepancy may be explained by the frequent discordance between mRNA and protein expression as pointed out earlier in the introduction, very low HIF-1α levels below LC-MS detection sensitivity, or differential half-life dynamics between P4HA1 and HIF-1α proteins.

In this study, we found high P4HA1 protein expression as an independent poor prognostic factor for early stage CRC, especially for the MSS subtype, using deep Fourier transform mass spectrometric proteomic discovery combined with immunohistochemical and clinicopathological validation in a total cohort of 712 patients. Early stage CRC presents frequent challenges in clinical patient management in that it is currently impossible to predict which patients will have aggressive disease and thus benefit the most from intensive adjuvant chemotherapy vs. those patients who will have less aggressive disease and benefit from surgery alone. Our current study focused on outcomes of patients with early stage CRC who were treated with surgery alone. Future work will look at the influence of adjuvant therapy on survival and whether P4HA1 protein expression renders patients more or less sensitive to certain adjuvant regimes. In addition, the MSS subgroup of CRC has been lacking prognostic biomarkers that would risk-stratify this type of CRC. Our discovery of P4HA1 outcome stratification in early stage CRC and, in particular, its MSS subtype, may provide an avenue for early stage CRC risk prognosis and thus improve cancer treatment outcomes by tailoring follow-up frequency and adjuvant therapy intensity.

## MATERIALS AND METHODS

### Fresh frozen tissue selection

For the initial proteomic discovery of protein biomarkers, we selected 22 CRC cases from Memorial Sloan Cancer Center with the tissue sample criteria of (i) high tumor content (>50%), (ii) no gross necrosis, and (iii) low blood contamination based on careful histologic examination of frozen sections prepared from each sample. Matched pairs of fresh frozen tumor tissue and benign colonic mucosa away from the cancer (carefully stripped without muscularis propria) were retrieved from the liquid nitrogen repository. Two gastrointestinal pathologists (AT and MHR) reviewed and verified histologic slides, diagnoses, and quality of all tissues. The study had been approved by the Institutional Review Board of Memorial Sloan Kettering Cancer Center.

### Validation cohorts

Validation studies were carried out with a cohort of 599 cases of early stage (AJCC stages I or II) CRC and another cohort of 91 cases of late stage (AJCC stages III or IV) CRC. All cases were from a single institution (Memorial Sloan Kettering Cancer Center) and had been resected between 1981 and 2010 (permitting long clinical follow-up). Clinical data including patient age, treatment history, and recurrence/survival status were retrieved from electronic medical records. Patients in the early stage cohort were selected to have undergone surgery only (with no adjuvant therapy) to make outcome data optimally comparable and not confounded by adjuvant therapy regimen heterogeneity (during cohort accrual and follow-up). For tissue microarrays, three separate 2-mm tissue cores each from tumor or normal mucosa were drilled out from each donor paraffin block and transferred to tissue array blocks using a robotic TMA arrayer (TMA Grand Master, 3DHistech). Tumor and normal areas were selected based on rigorous review of individual histologic slides for each donor block and electronic image-based coring target area selection in the TMA Grand Master software.

### Tissue proteome extraction

Samples of 5 mg of frozen tissue were thawed on ice and lysed with 200 μl lysis buffer containing 8 M urea, 0.1 M ammonium bicarbonate, phosphatase inhibitors 2 and 3 (Sigma), and protease inhibitors (Roche). The tissue mixture was homogenized with 12 cycles of 1-min sonication at 120 W power (FB120, Fisher Scientific) and intermittent cooling. After centrifugation at 14,000 g for 30 min at 4 °C, the supernatant which contains all soluble proteins was collected. The protein concentration was determined by a BCA assay (Pierce), and extracted proteomes were stored at –80 °C until further analysis.

### In-solution protein digestion

Aliquots of 50 μg of the lysate proteomes were reduced with 5 mM dithiothreitol at 56 °C for 30 min and then cooled to room temperate. The reduced proteins were alkylated with 11 mM iodoacetamide at room temperature for 30 min in the dark. The protein solution was diluted 6-fold with 50 mM ammonium bicarbonate and digested with trypsin and Lys-C (0.2 μg/μl, both from Promega) at 1:50 (w/w) at 37 °C for 12 h. The digestion was stopped by the addition of trifluoroacetic acid to a final concentration of 1%. The mixture was centrifuged at 14,000 g for 10 min at room temperature. The clear supernatant was collected and desalted on a C_18_ StageTip (lab-made). Desalted peptides were dried in a SpeedVac vacuum concentrator and re-dissolved in 10–15 μl of 3% acetonitrile/0.1% formic acid and stored at –20 °C.

### Proteomic analysis

Desalted peptides, approximately 1 μg, were injected into a 50-cm C_18_ capillary column mounted to an Easy-nLC 1200 system coupled to an Orbitrap Fusion Lumos mass spectrometer (Thermo Scientific). Peptides were eluted over a 200-min gradient in 2–35% buffer B (0.1% (v/v) formic acid, 100% acetonitrile) and buffer A (0.1% formic acid, 100% HPLC-grade water) at a flow rate of 300 nl/min. MS data were acquired with an automatic switch between a full scan and 10 data-dependent MS/MS scans. The target value for full-scan MS spectra was 1 × 10^6^ charges in the 375–1500 m/z range with a maximum injection time of 50 ms and a resolution of 60,000 at 200 m/z in profile mode. Isolation of precursors was performed with a window of 1.4 m/z. Precursors were fragmented by higher-energy C-trap dissociation with a normalized collision energy of 30 eV. MS/MS scans were acquired at a resolution of 15,000 at 200 m/z with an ion target value of 5 × 10^4^, maximum injection time of 100 ms, and dynamic exclusion for 15 s in centroid mode.

### Protein sequencing data analysis

Label-free protein quantification was carried out with MaxQuant (version 1.6.4.0) and the Andromeda search engine [31, 32]. The first and the main maximum precursor mass tolerances were set to 20 and 6 ppm, respectively. The reference human proteome database was downloaded from UniProt (with updates up to Sept. 2018). The search assumed trypsin and Lys-C digestions with up to 2 missed cleavages. A minimum of 1 peptide was required for protein identification, but 2 peptides were required to calculate a protein level ratio. The modifications used as variable modifications for protein identification and quantification included oxidation of methionine, acetylation of the protein N-terminus, phosphorylation of serine, threonine, and tyrosine residues, and deamidation of glutamine and asparagine. Significantly up-regulated and down-regulated proteins were identified with Perseus software [33, 34]. Enrichment analysis of GO terms and KEGG pathways was carried out with STRING [35]. Protein domain analysis was conducted with the SMART (Simple Modular Architecture Research Tool) through STRING [36].

### Immunohistochemistry (IHC)

P4HA1 expression was determined with P4HA1-specific antibodies (HPA026593, 1:2,000 dilution, Atlas Antibodies) on a Ventana BenchMark XT with OptiView DAB detection (Roche). HPA026593 has been validated as part of the Human Protein Atlas project (https://www.proteinatlas.org/ENSG00000122884-P4HA1/antibody) by peptide array, Western blotting, capture-MS, IHC, and immunocytochemistry. IHC results were scored by a semi-quantitative approach. Cytoplasmic staining intensity of individual tumor cells was determined and assigned intensities of 0, 1+, 2+, or 3+ (averaged across 3 independent tissue cores per case). The total weighted IHC score (IHC H-score) of a sample was calculated by multiplying the expression intensity of individual tumor areas (score, 0-3+) by their relative contribution (0-100%) to total tumor area and adding these to yield a total weighted sum. The IHC H-scores thus have a theoretical range of 0 to 300. Scoring of all tissue samples was independently performed by two pathologists. In cases of discrepancies in immunohistochemical assessment between the two pathologists, the cases were reviewed by them together and a consensus score was determined.

### Statistical analyses

Categorical variables were compared using Fisher’s exact test. Numerical values were analyzed by the Mann-Whitney U test. Survival analyses were performed using the Kaplan-Meier method and compared by a log-rank test. Multivariate analyses of prognostic factors was performed with logistic regression models by using factors that showed significant univariate differences (*p* < 0.05). A backward elimination method was used with a threshold of *p* = 0.05 to select variables for the final model. Statistical analyses were performed with JMP Pro 14 (SAS). All statistical analyses were considered significant with *p* < 0.05.

## SUPPLEMENTARY MATERIALS



## Abbreviations

CRC: colorectal cancer
IHC: immunohistochemistry
LC-MS: liquid chromatography-mass spectrometry
DFS: disease-free survival
OS: overall survival
