# Supplementary material for: Prolyl 4-hydroxylase alpha 1 protein expression risk-stratifies early stage colorectal cancer
Source: Oncotarget. 2020 Feb 25;11(8):813–24. doi: 10.18632/oncotarget.27491 (PMC7055541; doi:10.18632/oncotarget.27491)
Supplement: Supplementary file 1 [file oncotarget-11-813-s001.pdf]

# Prolyl 4-hydroxylase alpha 1 protein expression risk-stratifies early stage colorectal cancer

## SUPPLEMENTARY MATERIALS

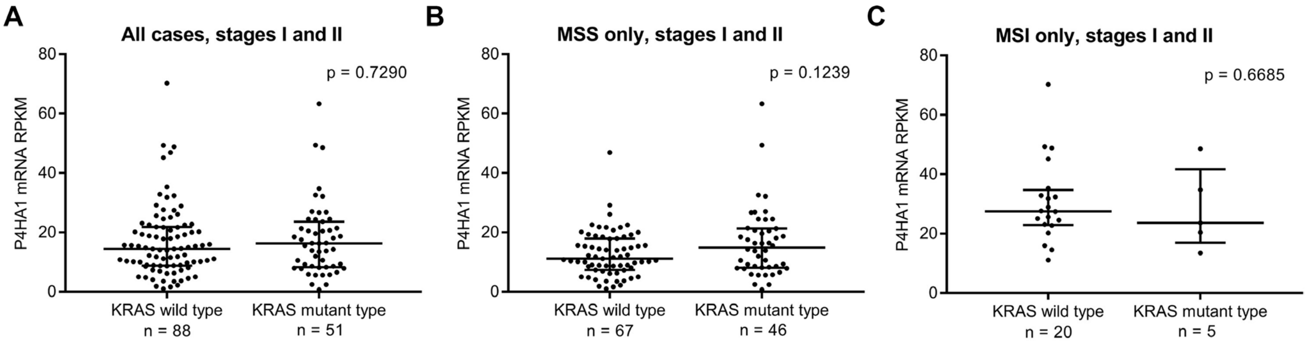


**Supplementary Figure 1: TCGA CRC dataset analysis focusing on KRAS gene mutation status.** *P4HA1* mRNA expression abundance as a function of *KRAS* status is shown. (**A**) All early stage cases. (**B**) Only MSS early stage cases. (**C**) Only MSI early stage cases. Note that there is no evidence of a dependence of the two variables. RPKM, reads per kilobase of transcript per million mapped reads.

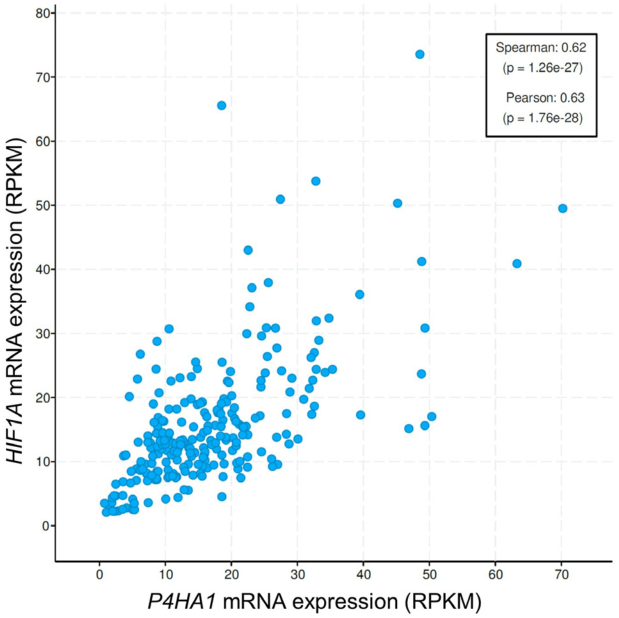


**Supplementary Figure 2: TCGA CRC dataset analysis focusing on HIF-1α expression.** Positive correlation between *P4HA1* mRNA expression abundance and *HIF-1α* mRNA expression abundance is shown. RPKM, reads per kilobase of transcript per million mapped reads.

**Supplementary Table 1: Clinicopathological characteristics of the late stage CRC cohort**

| Feature | P4HA1 (*n* = 91) | | *p*-value* |
|---|---|---|---|
| | Low | High | |
| Total | 53 (58.2%) | 38 (41.8%) | |
| Gender | | | 0.5234 |
| Male | 27 (62.8%) | 16 (37.2%) | |
| Female | 26 (54.2%) | 22 (45.8%) | |
| Age (years) | | | 0.0636 |
| ≤65 | 51 (61.5%) | 32 (38.5%) | |
| >65 | 2 (25.0%) | 6 (75.0%) | |
| Histology | | | 0.4826 |
| Mucinous | 4 (44.4%) | 5 (55.6%) | |
| Not mucinous | 49 (59.8%) | 33 (40.2%) | |
| Tumor differentiation | | | >0.9999 |
| G1/G2 | 47 (58.0%) | 34 (42.0%) | |
| G3 | 6 (60.0%) | 4 (40.0%) | |
| Location | | | 0.0497 |
| Left | 37 (67.3%) | 18 (32.7%) | |
| Right | 16 (44.4%) | 20 (55.6%) | |
| TNM stage | | | >0.9999 |
| III | 38 (57.6%) | 28 (42.4%) | |
| IV | 15 (60.0%) | 10 (40.0%) | |
| MMR | | | 0.2280 |
| Intact (MSS) | 48 (60.8%) | 31 (39.2%) | |
| Lost (MSI) | 5 (41.7%) | 7 (58.3%) | |

MMR, mismatch repair; *Fisher's exact test.
